# Uniportal video-assisted thoracoscopic anatomical resection of the right anterior pulmonary segment in a 10-year-old child with congenital pulmonary airway malformation

**DOI:** 10.1186/s13019-023-02221-5

**Published:** 2023-06-03

**Authors:** Seha Ahn, Youngkyu Moon

**Affiliations:** Department of Thoracic & Cardiovascular Surgery, Eunpyeong St. Mary’s Hospital, College of Medicine, The Catholic University of Korea, 1021, Tongil-ro, Eunpyeong-gu, Seoul, 03312 Republic of Korea

**Keywords:** Uniportal VATS, Anatomical resection, Right anterior pulmonary segment, CPAM

## Abstract

Congenital pulmonary airway malformation (CPAM) is a very rare phenomenon subject to malignant transformation that requires surgical resection. In an asymptomatic 10-year-old girl, we identified a single cystic and consolidated lesion on computed tomography. This incidental finding was confined to anterior segment of lung in right upper lobe (RUL). Uniportal video-assisted thoracoscopic surgery (VATS) served to successfully achieve anterior segmentectomy, without chest tube placement. The surgical specimen confirmed features of CPAM, also showing acute and chronic inflammation with abscess formation. Once the surgical mainstay for such lesions, open lobectomy is now under challenge by thoracoscopic technique, port-reduction methods, and a lung-preserving strategy. Herein, we have shown uniportal VATS anatomical resection of right anterior pulmonary segment to be a viable option for a 10-year-old child with CPAM confined to a single lung segment.

## Introduction

Congenital pulmonary airway malformation (CPAM), previously known as congenital cystic adenomatoid malformation (CCAM), is a very rare developmental anomaly of the lower respiratory tract [1, 2]. Its presentation is variable, although diagnosis during second trimester is customary, based on routine prenatal ultrasound examination [3]. Most neonates with prenatally determined CPAMs are asymptomatic at birth [4]; although they may later develop symptoms (cough, shortness of breath, or fever) or related conditions (pneumothorax, pneumonia, recurrent pulmonary infections, or malignancy).

At present, there are controversies about the optimal timing and extent of the resection in patients with asymptomatic CPAM. When performing surgery on these patients, the lesions were resected early, within the first six months of life [5]. There are advantages to surgical resection in these patients, as infection rates increase over time, making the operation more difficult and eliminating the risk of malignant transformation [2, 6]. Congenital lung anomalies typically have been resected via open thoracotomy. However, video-assisted thoracoscopic surgery (VATS) has grown in popularity, helping to shorten hospitalization stays and durations of chest tube placement [7, 8]. On the other hand, the thoracotomy group had shorter operative time than VATS [8]. Modifications of conventional VATS approach, particularly use of fewer access ports and avoidance of postoperative chest-tube drainage, have also reduced the stress of pulmonary resection [9, 10]. Still, the extent of surgery is currently under debate, deciding if full lobectomy is required, or whether segmental resection (to preserve lung tissue) will suffice [11, 12]. Herein, we describe a 10-year-old child undergoing uniportal (single-port) VATS S3 anterior segmentectomy, without drainage tube placement, for asymptomatic CPAM.

## Patient presentation

**Fig. 1:**
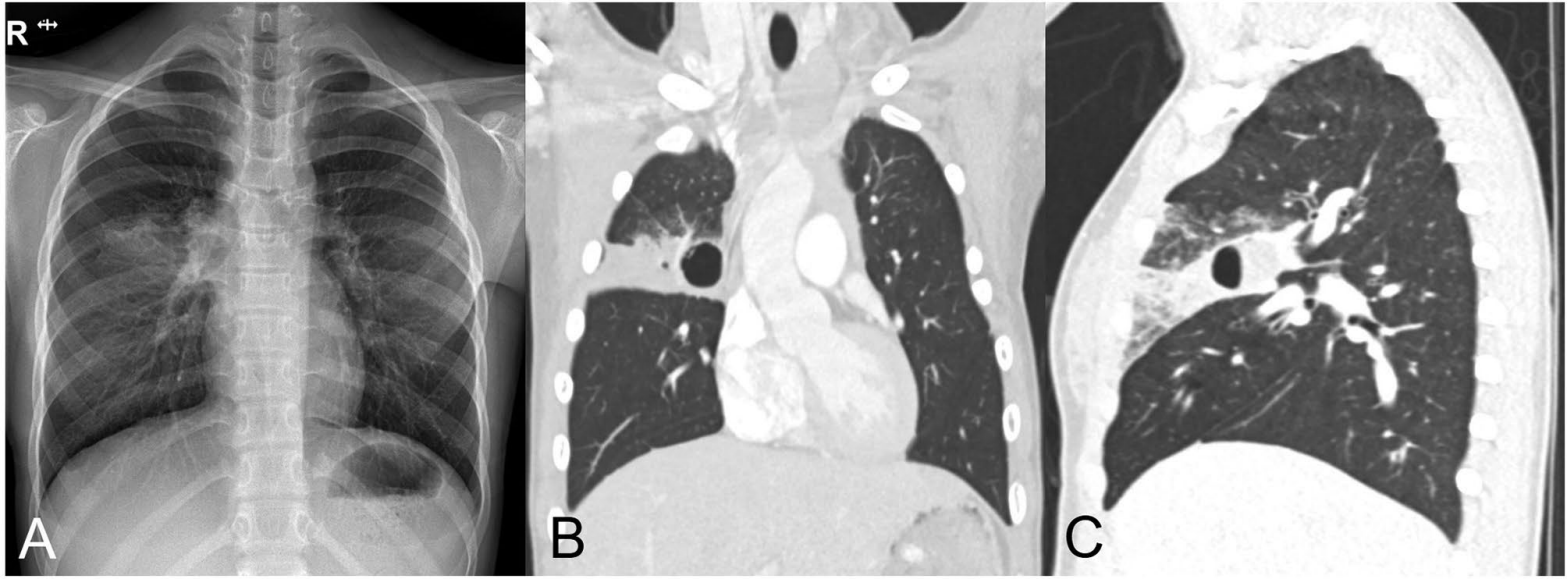
Preoperative imaging studies: (A) chest X-ray demonstrating cystic lesion and consolidation in right hilar area and (B, C) computed tomography of chest (coronal and sagittal views) with similar findings confined to anterior segment

A 10-year-old girl visited the Department of Otolaryngology as an outpatient, having sensed a mass/swelling on the left floor of mouth for 2 weeks. A history of bronchopulmonary dysplasia (BPD), linked to very low preterm birthweight, was acknowledged. There was a cystic lesion on floor of mouth, confirmed by computed tomography (CT) of the neck. The cyst (1.5 × 3.9 cm) occupied left anterior sublingual space, but a right hilar cystic lung lesion (2.3 × 1.5 cm) was also identified. She was referred to our Department of Thoracic and Cardiovascular Surgery to manage the lung lesion. On physical examination, the patient was asymptomatic and otherwise sound. Blood culture, oral culture, and urine culture were performed, and there was no growth of microorganisms. Therefore, no adjunctive antibiotics were administered. Chest X-ray again disclosed a cystic lesion and consolidation within right hilar region (Fig. 1A). CT of the chest further defined the consolidation, situated in anterior segment of right upper lobe (RUL) and harboring an internal cystic space (2.4 cm) with air-fluid level. Overall findings suggested a congenital cystic growth complicated by pneumonia (Fig. 1B, C).

**Fig. 2:**
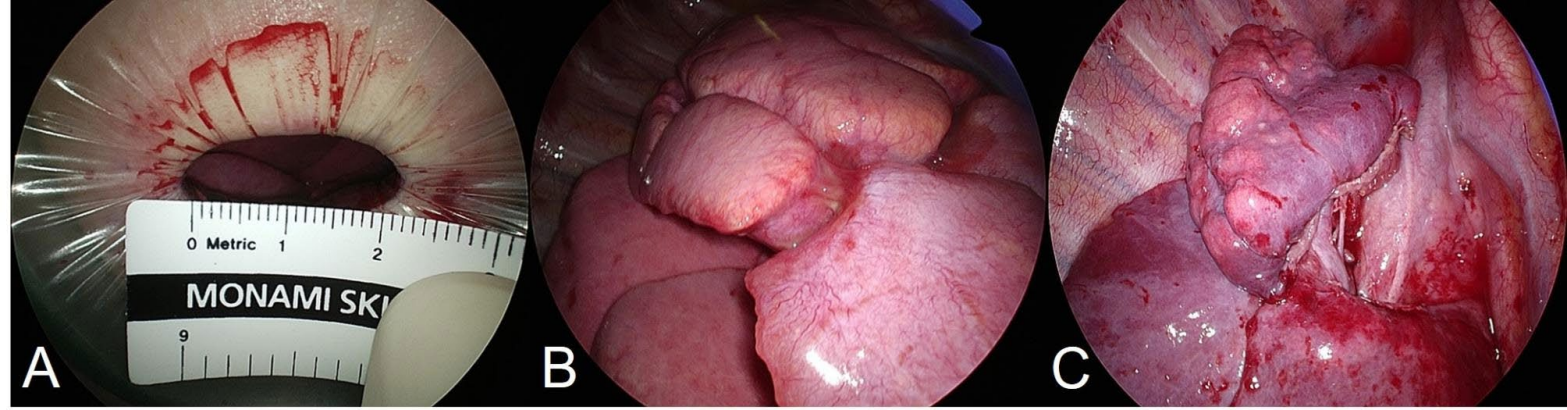
(A) Small working incision (2.3 cm) covered by wound protector. (B, C) Surgical view before and after uniportal VATS RUL anterior segmentectomy

A decision was made to conduct the surgery jointly, having both specialties participate. Under general anesthesia, lung-sparing resection was performed through uniportal VATS, hoping to to reduce surgical stress and preserve normal parenchyma. The patient’s height was 152 cm and weight was 40 kg, so thoracoscopic access was considered possible. This was followed by resection of the ranula. The patient was placed in left lateral decubitus position, using a double-lumen endotracheal tube for single-lung ventilation. A small surgical incision (2.3 cm) was made in anterior axillary line at fourth intercostal space (Fig. 2A). The working port was covered by a small wound protector (W-Shield Retractor X-S; SNT Medical, Seoul, Korea), and a 5-mm, 30° scope was stationed at its upper rim by the surgical assistant. Various instruments (i.e., curved suction tip, grasping tools, and articulating endostaplers) were inserted through the single incision.

The anterior segment of RUL was generally consolidated, with focal abscess formation (Fig. 2B). Close inspection was done to ensure there were no cystic areas in adjacent apical and posterior segments. The first step was to open the minor fissure via tunnel technique. Initially, we dissected the major fissure to locate interlobar arteries (as a later exit point for stapling). Mediastinal pleura was subsequently opened to dissect between upper and middle lobar veins. Once the central vein was identified, a clamp was placed lateral to middle lobar artery to include central vein. The minor fissure was finally transected, using a 45-mm stapler with a purple load. Mediastinal pleura abutting upper lobar artery was quite dense, requiring careful dissection to expose the anterior segmental pulmonary artery. Both artery and vein were isolated and divided using silk ligatures. Next, thorough lymphadenectomy (levels 11–13) was undertaken, exposing the anterior segmental bronchus. Before proceeding, two-lung ventilation was initiated, checking for inflation of RUL apical and posterior segments. Thereafter, we divided anterior segmental bronchus (45-mm stapler with purple load) and injected indocyanine green (ICG) to mark the intersegmental plane. RUL anterior segmentectomy was thus completed by dividing along this plane, using three purple-loaded 45-mm staplers.

The resected specimen was placed in an endocatch bag to retrieve it through the working port. No diseased areas (consolidated and cystic lesions) were found in the remaining apical and posterior segments of RUL, right middle lobe and right lower lobe of the thoracoscopic field of view (Fig. 2C). A water sealing method was performed to check for air leaks, and no air leaks were identified. Following irrigation, intercostal nerve blockade (bupivacaine, 1 mL) was performed at lower margins of third through seventh ribs. A 16-French chest tube was then inserted at the lower incisional edge, and the working incision was closed in layers. For skin closure, unidirectional absorbable barbed suture (V-Loc 180; Medtronic, Minneapolis, MN, USA) was used, leaving a thread attached. We connected the chest tube to a digital drainage system (DDS, Thopaz; Medela AG, Baar, Switzerland) with a suction pressure of -15 cm H_2_0, and the patient was moved from lateral decubitus to supine position for resecting the ranula.

**Fig. 3:**
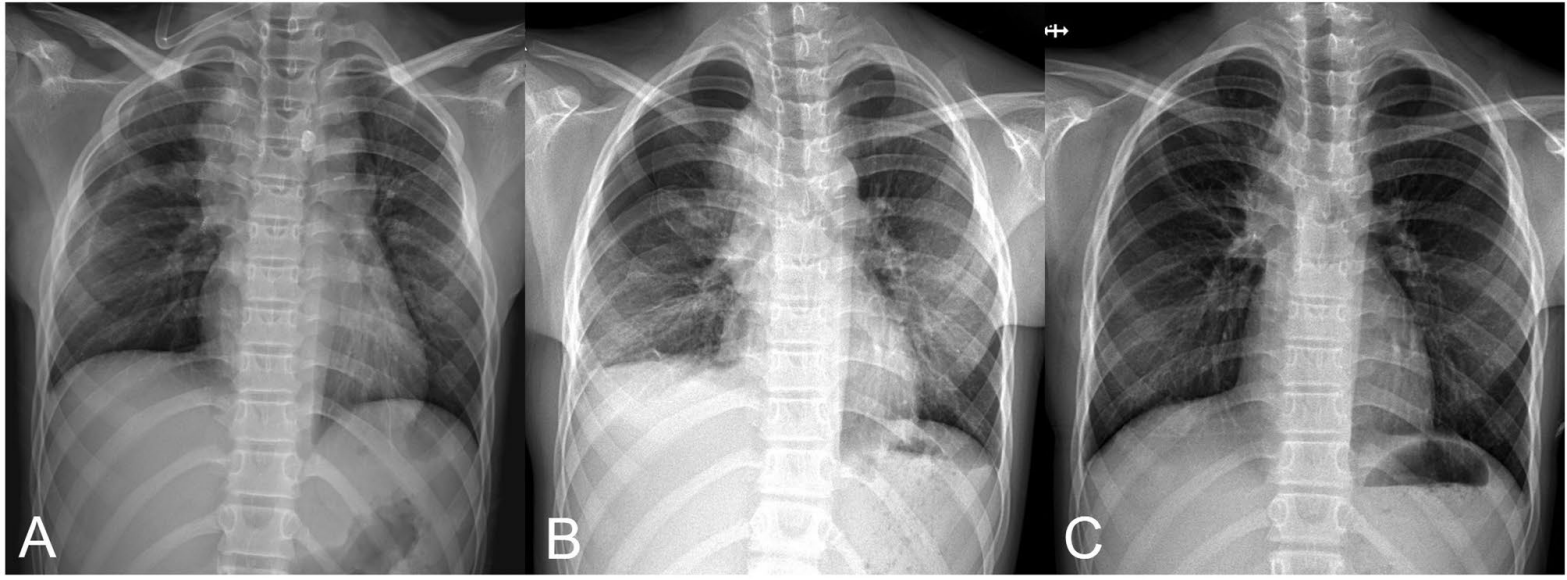
Postoperative chest radiographs: (A) clear upright anteroposterior film acquired in recovery room; (B) residual pneumothorax detected on postoperative Day 2; and (C) spontaneous resolution confirmed at first outpatient visit

After resecting the ranula, no airflow (0 mL/min) was checked with DDS, and the chest tube was removed in the operating room. The anesthesiologist provided an artificial deep inspiration (via bag valve mask) as this was done. To tighten closure of the working incision, the secured thread was pulled forward; and the remainder was cut, applying topical tissue adhesive (INDERMIL flexifuze; Connexicon Medical, Dublin, Ireland) to the site. The operation time was 215 min, and the anesthesia time was 240 min. Estimated blood loss was 50 cc. An upright anteroposterior chest X-ray obtained in the recovery room, 20 min after procedural completion, was clear (Fig. 3A). However, residual pneumothorax persisted on postoperative Day 2, without dyspnea or chest pain (Fig. 3B). Continuous oxygen was administered via a nasal cannula at 3 L/min. There was no further intervention during the hospitalization period, and the patient was discharged on postoperative Day 5. One week later, a follow-up chest X-ray at the first outpatient visit indicated full resolution (Fig. 3C).

**Fig. 4:**
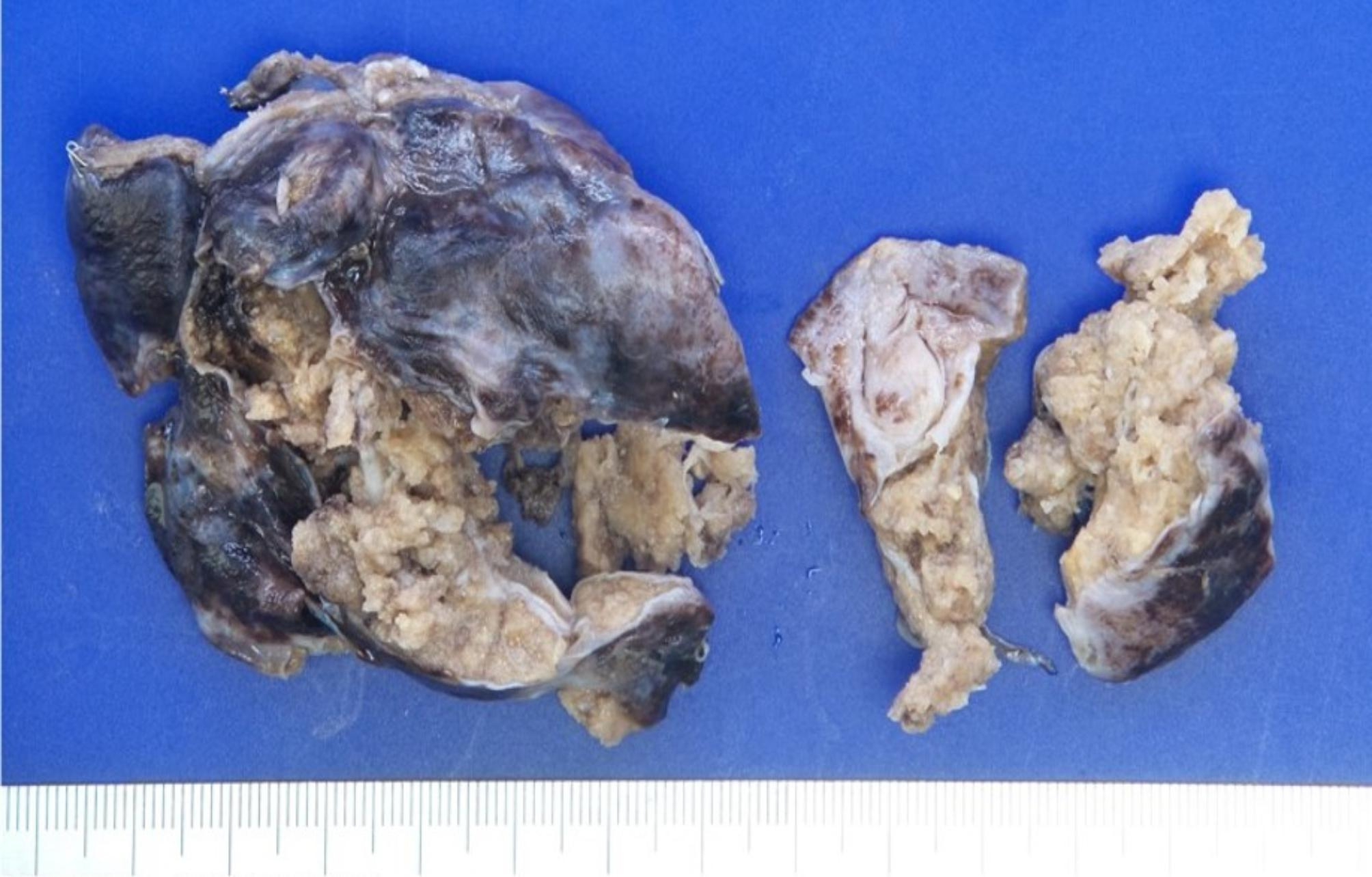
Gross photos of surgical specimen illustrating dusky external surface of resected right anterior segment and ragged abscess cavity

Gross photos of the resected lung specimen (7.5 × 5.2 × 3.2 cm) corroborated the radiographically depicted parenchymal abscess (Fig. 4). CPAM with acute and chronic inflammation and abscess formation was confirmed by the final pathology report.

## Discussion

CPAMs (formerly CCAMs) are very rare developmental anomalies ranging in incidence from 1 in 11,000–35,000 live births [13, 14]. The Stocker classification specifies three major types (I-III) of CCAM, based on cyst size and characteristics [15]. As of 2002, CPAM replaced CCAM in the nomenclature, and two more types of CPAM were designated. Type 0 arises from the trachea, and type 4 has alveolar and distal acinar origins [16, 17]. Type 1 accounts for most (60–70%) of such lesions, showing variably sized cysts (1–10 cm) of which at least one is dominant (> 2 cm across). Adjacent parenchyma is relatively normal, and in a majority of cases, only one lobe of lung is involved. Malignant transformation has been reported in CPAM types 1–4 [17]. In fact, respiratory symptoms and related conditions (pneumothorax, pneumonia, recurrent pulmonary infections, or malignancy) may be later developments in prenatally diagnosed infants, who are largely asymptomatic at birth [4]. We fortuitously discovered a single cystic lesion and a consolidated area in RUL (anterior segment) upon neck CT of our patient. Despite her lack of symptoms, clinical suspicion of CPAM prompted further examination and subsequent surgical intervention.

Many authorities presently recommend surgical resection of these defects to eliminate the risk of developing malignancies, such as pleuropulmonary blastoma or bronchioalveolar carcinoma [6, 13, 17]. Ultimately, our patient successfully underwent uniportal VATS S3 anterior segmentectomy, without drainage tube placement. Uniportal access was feasible, because only mediastinal pleura (in the vicinity of RUL pulmonary artery) showed severe adhesions. Also, a VATS approach was chosen for its reputed benefits, namely reduced durations of hospital stay and chest tube placement and mitigation of thoracotomy-related morbidity risk [7, 18, 19]. In a growing child, thoracotomy incision may result in shoulder girdle weakness, chest deformities, or scoliosis [20]. Controversy over the extent of surgery in instances of CPAM is ongoing, specifically whether removal of an entire lobe or a segment only (to preserve lung tissue) is appropriate [11]. Recent reports have shown that in patients with small and asymptomatic lesions, anatomic segmentectomy, as opposed to traditional lobectomy, does not heighten chances of residual or recurrent disease [12, 19, 21].

In this particular case, where the cystic/consolidated lesion of RUL was well confined, anterior segmentectomy was a viable option. Pairawan and his colleagues recently published a case report of an anatomical superior segmentectomy for a patient with large CPAM without the use of ICG[12]. In our case, the injection of ICG certainly helped, and we were able to clearly delineate the intersegmental plane without difficulty [22]. Anterior segmentectomy is ordinarily considered a complex procedure, but it is readily achievable by adopting a tunneling technique to divide the minor fissure [22, 23]. We also abandoned chest tube drainage, given a zero-level DDS airflow reading after resection of ranula (another 1-hour interval following lung surgery). DDS allowed us to precisely time chest-tube removal while providing constant negative pleural pressure [24]. Although residual pneumothorax persisted on postoperative Day 2, the patient was asymptomatic, and it had resolved spontaneously by the first outpatient visit.

## Conclusion

In summary, uniportal VATS anatomical resection of right anterior pulmonary segment is a valid therapeutic approach, along with other surgical options (i.e., multiport VATS or open lobectomy), for a 10-year-old child with CPAM confined to a single lung segment.

## Data Availability

The data underlying this article will be shared on reasonable request to the corresponding author.

## Abbreviations

CPAM: Congenital pulmonary airway malformation
RUL: Right upper lobe
VATS: Video-assisted thoracoscopic surgery
CCAM: Congenital cystic adenomatoid malformation
BPD: Bronchopulmonary dysplasia
CT: Computed tomography
ICG: Indocyanine green
DDS: Digital drainage system
